# Insulin Protects Pancreatic Acinar Cells from Palmitoleic Acid-induced Cellular Injury*

**DOI:** 10.1074/jbc.M114.589440

**Published:** 2014-07-03

**Authors:** Aysha Samad, Andrew James, James Wong, Parini Mankad, John Whitehouse, Waseema Patel, Marta Alves-Simoes, Ajith K. Siriwardena, Jason I. E. Bruce

**Affiliations:** From the ‡ Faculty of Life Sciences, The University of Manchester, M13 9NT Manchester and; the § Hepatobiliary Surgery Unit, Manchester Royal Infirmary, M13 9WL Manchester, United Kingdom

**Keywords:** Calcium, Calcium ATPase, Calcium Transport, Insulin, Pancreas, PMCA, Pancreatitis

## Abstract

Acute pancreatitis is a serious and sometimes fatal inflammatory disease where the pancreas digests itself. The non-oxidative ethanol metabolites palmitoleic acid (POA) and POA-ethylester (POAEE) are reported to induce pancreatitis caused by impaired mitochondrial metabolism, cytosolic Ca^2+^ ([Ca^2+^]*_i_*) overload and necrosis of pancreatic acinar cells. Metabolism and [Ca^2+^]*_i_* are linked critically by the ATP-driven plasma membrane Ca^2+^-ATPase (PMCA) important for maintaining low resting [Ca^2+^]*_i_*. The aim of the current study was to test the protective effects of insulin on cellular injury induced by the pancreatitis-inducing agents, ethanol, POA, and POAEE. Rat pancreatic acinar cells were isolated by collagenase digestion and [Ca^2+^]*_i_* was measured by fura-2 imaging. An *in situ* [Ca^2+^]*_i_* clearance assay was used to assess PMCA activity. Magnesium green (MgGreen) and a luciferase-based ATP kit were used to assess cellular ATP depletion. Ethanol (100 mm) and POAEE (100 μm) induced a small but irreversible Ca^2+^ overload response but had no significant effect on PMCA activity. POA (50–100 μm) induced a robust Ca^2+^ overload, ATP depletion, inhibited PMCA activity, and consequently induced necrosis. Insulin pretreatment (100 nm for 30 min) prevented the POA-induced Ca^2+^ overload, ATP depletion, inhibition of the PMCA, and necrosis. Moreover, the insulin-mediated protection of the POA-induced Ca^2+^ overload was partially prevented by the phosphoinositide-3-kinase (PI3K) inhibitor, LY294002. These data provide the first evidence that insulin directly protects pancreatic acinar cell injury induced by *bona fide* pancreatitis-inducing agents, such as POA. This may have important therapeutic implications for the treatment of pancreatitis.

## Teaser

**Background:** Palmitoleic acid is a major pancreatitis-inducing agent.

**Results:** Insulin protected cells from palmitoleic acid (POA)-induced ATP depletion, inhibition of the plasma membrane calcium pump (PMCA), cytotoxic Ca^2+^ overload and necrosis.

**Conclusion:** Insulin protects against acinar cell injury induced by pancreatitis-inducing agents.

**Significance:** This provides an important therapeutic strategy for treating pancreatitis with insulin therapy.

## Introduction

Acute pancreatitis (AP)^4^ is an inflammatory disease caused by autodigestion, resulting in pancreatic necrosis, systemic inflammation, and in severe cases multiple organ failure (1–5). There is no imminent cure, and the overall disease-related mortality, especially in severe disease, is 30% (5–7). The major causes of acute pancreatitis include bile acids from gall stones and ethanol metabolites from excessive alcohol consumption (2, 3, 6). Impairment of mitochondrial metabolism and cytosolic Ca^2+^ ([Ca^2+^]*_i_*) overload in pancreatic acinar cells have been implicated as the cardinal pathological events common to most forms of pancreatitis, regardless of the precise causative factor (3). Metabolism and [Ca^2+^]*_i_* are linked in various ways, perhaps most critically by the ATP-driven plasma membrane Ca^2+^-ATPase (PMCA), which provides a final common path for cells to restore [Ca^2+^]*_i_* during cellular injury (8). Therefore, restoration of metabolism and protection of PMCA function represents an attractive and untapped locus for therapeutic intervention.

One way in which this might be achieved is by treatment with insulin. Evidence suggests that insulin may ameliorate the course of clinical acute pancreatitis (9–11) and protect against experimentally induced pancreatitis (12–16). In addition, ∼50% of diabetic patients exhibit pancreatic exocrine lesions characteristic of chronic pancreatitis (17), and type 2 diabetics have an ∼3-fold increased risk of developing acute pancreatitis (18). It is not clear whether protection against acute pancreatitis produced by insulin is due to a direct effect on acinar cells, modification of the systemic inflammatory response (19, 20) or due to tight glycaemic control, which reduces the chance of sepsis (21).

However, our previous study provided the first evidence that insulin treatment directly protects acinar cells from cellular injury induced by oxidative stress (22). Although oxidants such as H_2_O_2_ mimic many of the cellular events during pancreatitis, this is not the most patho-physiologically relevant pancreatitis-inducing agent and might have additional effects not observed during pancreatitis. Therefore, the aim of the current study was to test the protective effects of insulin on pancreatic acinar injury induced by *bona fide* pancreatitis-inducing agents, such as ethanol and ethanol/fatty acid metabolites, including palmitoleic acid ethylesters (POEE) and palmitoleic acid (POA) (23).

The results show that in isolated rat pancreatic acinar cells, POA was more cytotoxic than either POAEE or ethanol itself, consistent with previous studies (24–29). POA induced a cytotoxic irreversible Ca^2+^ overload and necrotic cell death that was due to ATP depletion and inhibition of the PMCA. Consistent with our previous study, insulin pre-treatment either markedly attenuated or prevented all these POA-induced responses. Moreover, the protective effects of insulin were due in part to activation of PI3K/Akt. These data provide further evidence that insulin directly protects pancreatic acinar cells against *bona fide* pancreatitis-inducing agents.

## EXPERIMENTAL PROCEDURES

### 

#### 

##### Cell Isolation

Pancreatic acinar cells from Sprague-Dawley rats were isolated by collagenase digestion as previously described (30, 31). Briefly, the pancreas was quickly dissected from the rat and repeatedly injected with ice cold solution containing 0.15 mg/ml collagenase-P (Roche Diagnostics), 0.12 mg/ml soybean trypsin inhibitor (Sigma), and 1% (*w*/*v*) bovine serum albumin (BSA, Fraction V, Sigma) in a HEPES-buffered physiological saline solution (HEPES-PSS; composition in mm: 137 Na^+^, 4.7 K^+^ 0.56 Mg^2+^, 1.28 Ca^2+^, 143.5 Cl^−^, 1 HPO_4_^2−^, and 10 HEPES, with a pH of 7.4). The inflated pancreas was then rapidly chopped with fine scissors and allowed to incubate for 30 min, replacing the solution after 15 min. Tissue clusters were then incubated with collagenase-free, Ca^2+^-free HEPES-PSS, containing 1 mm EDTA, for a further 5 min. This was followed by trituration to break up the tissue into small clusters, which were then filtered through a nylon mesh and centrifuged in 4% BSA HEPES-PSS (*w*/*v*) for 5 min at 300 × *g*. Following digestion, cells were resuspended in HEPES-PSS containing 0.1% BSA and kept on ice until use. Animals were humanely killed by an approved method as set out in Schedule 1 of the UK Animals Scientific Procedures Act 1986 (Certificate of Designation No 50/2506).

##### Imaging of Fura-2 Fluorescence

Pancreatic acinar cells were loaded with 4 μm fura-2-AM (Invitrogen, Paisley, UK) for 30 min at room temperature in HEPES-PSS as previously described (30). Fura-2-loaded cells were imaged using an identical microscope/imaging system to previous studies (30). Briefly this comprised a Nikon TE2000S microscope with ×40 oil immersion SFluor objective lens, CoolSNAP HQ CCD camera (Photometrics, Tucson, AZ) and Cairn monochromator (Cairn Research, Kent, UK). Image acquisition and analysis was controlled by MetaFluor imaging software (Molecular Devices, Downington, PA). Background-subtracted 340-nm and 380-nm fluorescence images were captured with 50 ms exposure and 3 × 3 binning every 5 s. The fura-2 fluorescence was calibrated into “estimated” [Ca^2+^]*_i_* using the well established method as previously described (31). For all fluorescence imaging experiments, cells were perfused with a HPO_4_^2−^-free HEPES-PSS to avoid precipitation with high concentrations of Ca^2+^. All experiments were carried out at room temperature (20–22 °C).

##### In Situ Ca^2+^ Clearance Assay

Cells were treated with the sarco/endoplasmic reticulum Ca^2+^-ATPase (SERCA) inhibitor, cyclopiazonic acid (CPA) in zero external Ca^2+^. This induces ER Ca^2+^ depletion and activation of store-operated Ca^2+^ entry (SOCE). Addition of 20 mm external Ca^2+^ to cells therefore results in a rapid increase in [Ca^2+^]*_I_*, which reaches a short-lived steady state. The subsequent removal of external Ca^2+^ (0 Ca^2+^, 1 mm EGTA) causes a rapid [Ca^2+^]*_i_* clearance due predominantly to PMCA activity. Repeated Ca^2+^ influx-efflux phases allow test reagents to be applied during the second phase and compared with the first phase. [Ca^2+^]*_i_* clearance rate (and thus PMCA activity) was quantified by measuring the linear rate from a standardized value of [Ca^2+^]*_i_* during the second clearance phase and normalized to the corresponding linear rate (over the initial 60 s) during the first clearance phase (30). Measuring the linear rate was found to be more appropriate than fitting to a single exponential decay, because [Ca^2+^]*_i_* did not always reach the same asymptotic baseline. This normalized rate with various test reagents was compared with corresponding time-matched control experiments.

##### Measurement of Cellular ATP

ATP depletion was determined using two complementary techniques; magnesium green (MgGreen) and fire-fly luciferase (ViaLight® Plus kit; Lonza, Rockland, ME) as previously described (22). Cells were treated with or without 100 nm insulin for 20 min, followed by 100 μm POA for a further 20 min before treatment with an ATP depletion mixture (4 μm CCCP, 500 μm bromopyruvate, 10 μm oligomycin, 2 mm iodoacetate, and 100 μm carbachol) to induce maximum ATP depletion. All responses were normalized to the maximum ATP depletion induced by the ATP depletion mixture (22). For the ViaLight® Plus kit cells were treated with or without 100 nm insulin for 20 min, followed by treatment with or without various concentrations of POA (10–300 μm) for a further 20 min before treatment with or without the ATP depletion mixture for a further 20 min. The total luminescence count of the ATP-depletion mixture was subtracted from each corresponding assay condition prior to normalization to the corresponding time-matched controls (%).

##### Cell Death Assays

Necrotic cell death was assessed using the cell impermeable nuclear-staining fluorescent dye, propidium iodide (PI). Pancreatic acinar cells were seeded into 96-well plates containing HEPES-PSS at an optimum density that was empirically determined to ensure maximum coverage of each well without compromising the ability to count the cells due to overcrowding. PI was added to each well from a ×100 stock to give a final concentration of 1.5 μm and was allowed to incubate for 30 min prior to the acquisition of images. Corresponding brightfield and fluorescent images (3–5 images per well) were captured using a ×20 objective lens at 5 × 5 binning. PI-stained cells were excited with 545 ± 10 nm excitation light using a Cairn Monochromator (Cairn Research, Kent, UK) and emitted light was separated from excitation light using a TRITC dichroic (Chroma, VA). Fluorescent images were captured onto a CoolSNAP HQ CCD camera (Photometrics). Image acquisition was controlled by MetaFluor imaging software (Molecular Devices) and TIF images analyzed using Image J.

In initial experiments total cell count was determined using the cell permeable nuclear staining fluorescent dyes, DAPI or Syto-16. This was done in an attempt to normalize PI-positive cell count (as a %) to the total cell count (either DAPI- or Syto-16-positive cells). However, it became apparent that both these dyes, routinely used to assess total cell count, did not always label every cell (either live or dead), which therefore under-estimated the total cell count and overestimated the % dead cell count. Therefore, total cell count was instead determined from brightfield images. Each field of view contained around 600–900 cells. Multiple fields of view (3–5) were taken for each well and % PI-positive cell count was averaged. For each cell preparation each treatment was performed in triplicate. Cells (total and PI-positive) were manually counted offline using the cell counter plugin within Image J. As a positive control, cells were treated with 0.1% Triton-X 100, which always gave 100% cell death confirming that the PI assay was working. Pancreatic acinar cells were treated with or without 100 μm POA for 30, 60, 120, or 180 min prior to image acquisition. The minimum period of 30 min was chosen as this was the minimum period required for optimum PI staining; therefore POA and PI were added at the same time for the 30-min period, whereas PI was added 30 min before the end of each other subsequent period. For insulin-treated cells 100 nm insulin was added 20 min prior to the addition of either POA and/or PI. Mean (± S.E.) % PI-positive cell count (% dead cells) was determined from five separate cell preparations.

##### Data Analysis and Experimental Design

Where possible a balanced experimental design was adopted, whereby responses to either POA, POA with insulin, or POA with insulin and LY293004 and corresponding time-matched controls were performed on the same day from the same cell preparation. Changes in resting [Ca^2+^]*_i_* were quantified using both the maximum change above resting [Ca^2+^]*_i_* and area under the curve (AUC) and responses compared using a one-way ANOVA with Bonferroni correction. For [Ca^2+^]*_i_* clearance experiments, a paired experimental design was adopted, in which two repeated Ca^2+^ clearance phases were elicited and the normalized linear [Ca^2+^]*_i_* clearance rate was compared with time matched control experiments using a Kruskal Wallis with post hoc Dunns test (30). For cell death assays different treatments were compared with corresponding time-matched controls or between treatments using a two-way ANOVA with Bonferroni correction. For all parameters, an “experimental average” was determined from several cells in each experiment, and these values were in turn averaged to give the true overall mean ± S.E. All data analysis was performed using Graphpad Prism 6 software.

## RESULTS

### 

#### 

##### Effect of Ethanol and Non-oxidative Ethanol/Fatty Acid Metabolites on Resting [Ca^2+^]_i_

**FIGURE 1.**
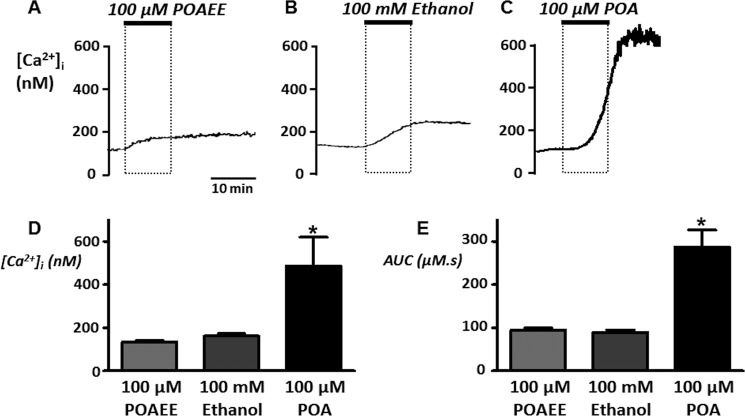
**Effects of ethanol and non-oxidative ethanol/fatty acid metabolites on resting [Ca^2+^]*_i_*.** Fura-2-loaded pancreatic acinar cells were treated with 100 μm POAEE (*A*), 100 mm ethanol (*B*), and 100 μm POA (*C*) for 10 min where indicated. Mean data (± S.E.) for maximum change in resting [Ca^2+^]*_i_* (*D*) and area under the curve (AUC, *E*) over the treatment and recovery period. *, *p* < 0.05 as determined by unpaired Student's *t* test.

Previous studies have shown that ethanol toxicity in mouse pancreatic acinar cells is due to the non-oxidative metabolites of ethanol and fatty acid, palmitoleic ethyl ester (POAEE), and palmitoleic acid (POA) (24, 25). To determine whether insulin has any protective effect against these pancreatitis-inducing agents it was necessary to confirm the effects of these agents on resting [Ca^2+^]*_i_*. In the present study in rat pancreatic acinar cells, both POAEE (100 μm) and ethanol (100 mm) caused a small but irreversible increase in resting [Ca^2+^]*_i_* by 134 ± 9 and 162 ± 12 nm, respectively (Fig. 1, *A*, *B*, and *D*) and AUC of 94 ± 6 and 89 ± 6 μm.s (Fig. 1*E*). POA (100 μm), on the other hand, caused a much greater irreversible increase in [Ca^2+^]*_i_* by 487 ± 40 nm (Fig. 1, *C* and *D*) and AUC to 247 ± 40 μm.s (Fig. 1, *C* and *E*), consistent with previous studies in mouse pancreatic acinar cells (24, 25).

##### Insulin Protects against POA-induced Ca^2+^ Overload

**FIGURE 2.**
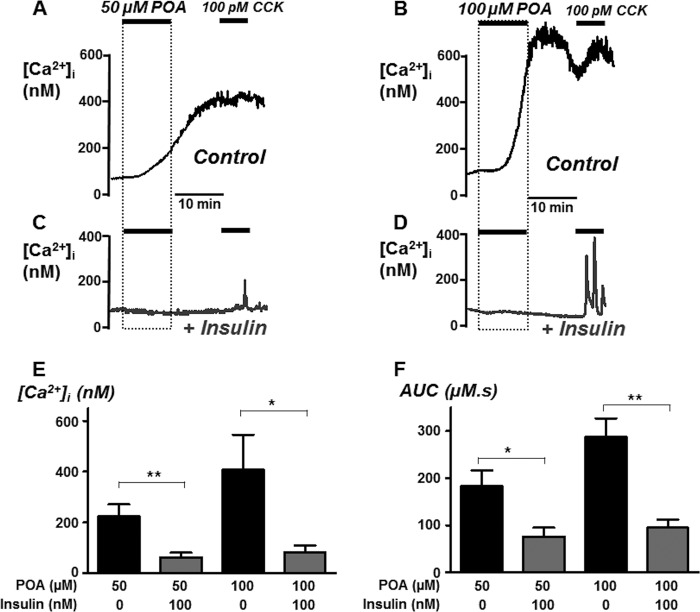
**Insulin protects against palmitoleic-induced Ca^2+^ overload.** Representative traces showing POA-induced responses (*A–D*) in untreated fura-2-loaded pancreatic acinar cells (*A* and *B*) and following pre-treatment with 100 nm insulin for 30 min (*C* and *D*). Overall mean data (± S.E.) showing maximum change in resting [Ca^2+^]*_i_* above baseline [Ca^2+^]*_i_* (*E*) and area under the curve (AUC; *F*) over the treatment and recovery period in the absence (*black box*) or following treatment with insulin (*gray box*). *, *p* < 0.05 and **, *p* < 0.01 as determined by unpaired Student's *t* test.

Because POA appeared to be the most cytotoxic pancreatitis-inducing agent tested, we next wanted to test whether insulin pre-incubation would prevent this POA-induced [Ca^2+^]*_i_* overload. Pancreatic acinar cells were pre-treated with or without 100 nm insulin for 30 min, followed by treatment with 50 or 100 μm POA for 10 min. Cells were then allowed to recover for a subsequent period of 10 min, followed by treatment with 100 pm CCK to test for irreversibility of the POA response and thus an indirect measure of cell viability (Fig. 2, *A–D*).

On average, 50 μm POA increased [Ca^2+^]*_i_* by 224 ± 48 nm (*n* = 6), which was reduced to 63 ± 18 nm in the presence of insulin (*n* = 6, *p* < 0.01 *versus* POA alone). Likewise, the POA-induced increase in AUC (183 ± 33 μm.s, *n* = 6) was also markedly reduced in insulin-treated cells (76 ± 19 μm.s, *n* = 6, *p* < 0.05). Similarly the 100 μm POA-induced increase in [Ca^2+^]*_i_* was reduced from 407 ± 139 nm (*n* = 6) to 83 ± 26 nm (*n* = 6, *p* < 0.05 *versus* control cells) by insulin. Likewise, AUC (287 ± 40 μm.s, *n* = 6) was also markedly reduced by insulin (95 ± 18 μm.s, *n* = 6, *p* < 0.001). Collectively, these data clearly show that insulin protects pancreatic acinar cells against POA-induced cytotoxic [Ca^2+^]*_i_* overload.

##### Insulin Protection of POA-induced Ca^2+^ Overload Is Partially Prevented by the PI3K Inhibitor, LY294002

**FIGURE 3.**
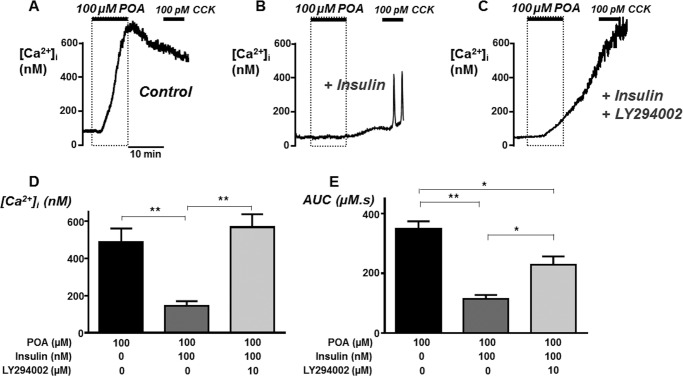
**Insulin protection of palmitoleic-induced Ca^2+^ overload was partially prevented by the PI3K inhibitor, LY294002.** Representative traces showing POA-induced responses (*A–C*) in untreated fura-2-loaded pancreatic acinar cells (*A*) and following pre-treatment with 100 nm insulin (*B*) or a combination of 100 nm insulin and 10 μm LY294002 for 30 min (*C*). Overall mean data (± S.E.) showing maximum change in [Ca^2+^]*_i_* above baseline [Ca^2+^]_i_ (*D*) and area under the curve (AUC; *E*) over the treatment and recovery period for POA alone (*black box*) or POA following treatment with insulin (*dark gray box*) or insulin and LY294002 (*light gray box*). *, *p* < 0.05 and **, *p* < 0.01 as determined by one-way ANOVA with Bonferroni correction.

Insulin couples to numerous downstream signaling pathways including activation of intrinsic receptor tyrosine kinase activity, MAPK and PI3K/Akt activity. However, most of the acute short-term effects that do not involve changes in gene expression are mediated through PI3K/Akt. To test whether the protective effects of insulin were due to activation of PI3K/Akt pathways, cells were pre-treated with insulin in combination with the PI3K inhibitor, LY294002 (10 μm) prior to treatment with 100 μm POA. Responses were then compared with parallel experiments performed on the same day in which cells were treated with either POA alone or POA with insulin pre-treatment (Fig. 3, *A* and *B*). This concentration of POA (100 μm) was chosen because it produced the most robust Ca^2+^ overload response and was almost completely abolished by insulin pre-treatment (Fig. 2). Our previous study showed that LY294002 had no effect on resting [Ca^2+^]*_i_* when applied alone (22), suggesting that inhibition of any constitutively active PI3K has no effect on resting [Ca^2+^]*_i_*. However, the combined treatment of cells with LY294002 (10 μm) and insulin (100 nm) appeared to partially reverse the protective effect of insulin (Fig. 3, *C*, *D*, and *E*). Specifically, the maximum increase in POA-induced [Ca^2+^]*_i_* with insulin and LY294002 (567 ± 69 nm, *n* = 6; Fig. 3, *C* and *D*) was completely reversed; there was no difference compared with POA alone (488 ± 73, *n* = 6; Fig. 3, *A* and *D*) but was significantly different from POA and insulin (143 ± 27, *n* = 6, *p* < 0.05; Fig. 3, *B* and *D*). However, the AUC for the POA-induced [Ca^2+^]*_i_* with insulin and LY294002 (229 ± 28 μm.s, *n* = 6; Fig. 3, *C* and *E*) was only partially reversed. The response was significantly lower than POA alone (348 ± 26 μm.s, *n* = 6; Fig. 3, *A* and *E*) and POA and insulin (115 ± 13 μm.s, *n* = 6, *p* < 0.05; Fig. 3, *B* and *E*). This lower AUC was due to a significant delay in the rise in [Ca^2+^]*_i_* even though this eventually resulted in a similar maximal increase in [Ca^2+^]*_i_*. Nevertheless, these data suggest that activation of PI3K/Akt is only partially responsible for the protective effects of insulin and that another parallel signaling pathway may also be responsible.

##### Insulin Prevents the POA-induced and Time-dependent Inhibition of the PMCA

**FIGURE 4.**
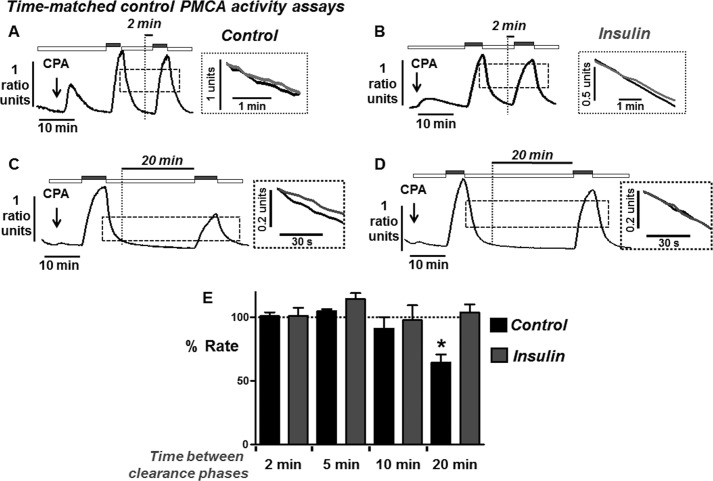
**Time-dependent effects on PMCA activity during time-matched control *in situ* [Ca^2+^]*_i_* clearance experiments.** Representative traces showing time-matched control *in situ* [Ca^2+^]*_i_* clearance experiments from untreated fura-2-loaded pancreatic acinar cells (*A* and *C*) and cell pre-treated with insulin (*B* and *D*) in which various amounts of time (2 or 20 min) were allowed between the first and second influx-clearance phases. Cells were treated with 30 μm CPA (*arrow*) in the absence of external Ca^2+^ with 1 mm EGTA (*white box*) or 20 mm Ca^2+^ (*gray box*) to induce the Ca^2+^ influx phase. Times between the first influx-clearance phase reaching a new baseline and addition of 20 mm Ca^2+^ to initiate the second influx-clearance phase included; 2 min (*n* = 5, *A*; mean data, *E*); 5 min (*n* = 5; mean data, *E*), 10 min (*n* = 5; mean data, *E*) and 20 min (*n* = 5, *C*; mean data, *E*). *Insets* for *A--D* represent expanded time-courses taken from the first (*gray*) and second clearance phases (*black*). Linear clearance rate measured over the initial 60 min from a standardized value was normalized to the initial clearance rate in each cell (% relative clearance). *E*, mean % relative clearance (± S.E.) during time-matched control *in situ* [Ca^2+^]*_i_* clearance in untreated cells (*black boxes*) and cell pre-treated with insulin (*gray boxes*). *, *p* < 0.05 statistical significance as determined by a Kurskal Wallis with post hoc Dunns test.

To test whether the protective effect of insulin on POA-induced Ca^2+^ overload was due to protection of PMCA activity, we utilized a similar *in situ* [Ca^2+^]*_i_* clearance assay to our previous studies (22, 30). As previously discussed and validated in our previous studies (22, 30), [Ca^2+^]*_i_* clearance under these conditions is almost exclusively due to PMCA activity. Moreover, the paired experimental design controls for both cell-to-cell, and time-dependent, differences in PMCA activity (see Fig. 4).

**FIGURE 5.**
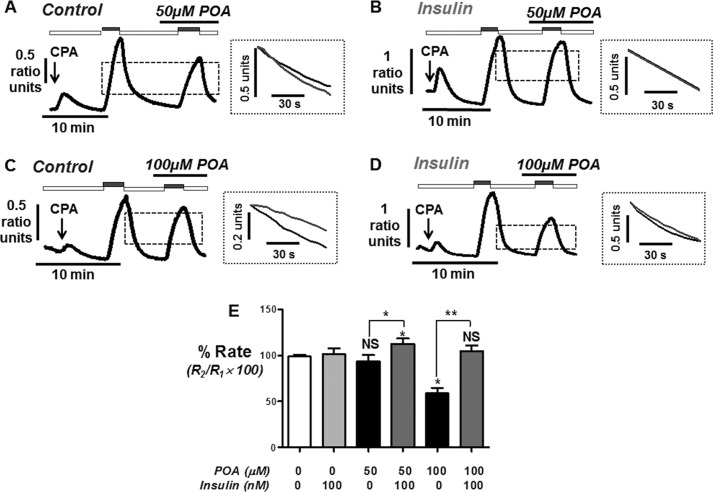
**Insulin protection against palmitoleic acid-induced inhibition of the PMCA during short duration (2 min) *in situ* [Ca^2+^]*_i_* clearance assays.** Representative traces showing the effect of POA (*A–D*) on *in situ* [Ca^2+^]*_i_* clearance in untreated (*A* and *C*) and insulin-treated cells (*B* and *D*). Cells were treated with 30 μm CPA (*arrow*) in the absence of external Ca^2+^ with 1 mm EGTA (*white bar*) or 20 mm Ca^2+^ (*gray bar*) to induce the Ca^2+^ influx phase. POA was added when Ca^2+^ recovered to baseline after the first influx-clearance phase for 2 min prior to re-addition of 20 mm Ca^2+^. *Inset* (*A–D*) shows superimposed expanded time-courses of first (*black trace*) and second clearance phase (*gray trace*) taken from the *dashed box* in *A--D*. Linear clearance rate (in the presence of POA) was normalized to the initial clearance rate in each cell (% relative clearance). *E*, mean % relative clearance (± S.E.) of corresponding time-matched control (*white box*), insulin treatment alone (*light gray box*), POA treatment (50 and 100 μm; *black box*), and POA with insulin (*dark gray box*). *, *p* < 0.05; **, *p* < 0.01 compared with corresponding time-matched control as determined by Kurskal Wallis with post hoc Dunns test.

Using this approach, the second clearance rate was found to be on average 99 ± 2% of the initial clearance rate in time-matched control experiments (*n* = 5; Figs. 4 and 5*E*). Initial experiments revealed that treatment with 50 μm POA during this second influx-clearance phase had no effect on clearance rate (94 ± 7%, *n* = 6, Fig. 5*A*). This seemed counterintuitive, since 50 μm POA induced a robust irreversible [Ca^2+^]*_i_* overload (Figs. 1*C* and 2*A*). However, the time taken to reach the maximum increase in [Ca^2+^]*_i_* was between 10 and 20 min (Figs. 1*C* and 2*A*), whereas POA was applied on average 2 min prior to the influx-clearance phase (Fig. 5*A*). We therefore reasoned that POA must be applied for longer periods prior to measuring its effects on [Ca^2+^]*_i_* clearance. To test this it was necessary to carry out a series of time-matched control experiments in which various periods of time (2, 5, 10, and 20 min) were allowed between the recovery of the first clearance phase and the start of the second influx-clearance phase (Fig. 4). These experiments were repeated following treatment with insulin (100 nm for 30 min; Fig. 4). Reassuringly, relative [Ca^2+^]*_i_* clearance rate remained remarkably constant after 2, 5, and 10 min both with or without insulin treatment (see mean data; Fig. 4*E*). However, after 20 min, [Ca^2+^]*_i_* clearance was significantly inhibited to 65 ± 6%, (*n* = 7, *p* < 0.05; Fig. 4, *C* and *E*). Interestingly, however, in cells pre-treated with insulin this did not happen and [Ca^2+^]*_i_* clearance was maintained even after 20 min (104 ± 7%, (*n* = 8; Fig. 4, *D* and *E*). This therefore suggests that insulin protects cells from the time-dependent inhibition of the PMCA.

**FIGURE 6.**
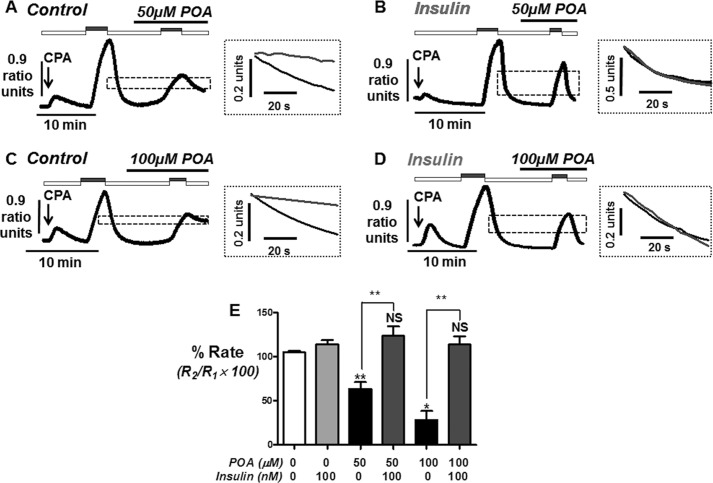
**Insulin protection against palmitoleic acid-induced inhibition of the PMCA during intermediate duration (5 min) *in situ* [Ca^2+^]*_i_* clearance assays.** Representative traces showing the effect of POA (*A–D*) on *in situ* [Ca^2+^]*_i_* clearance in untreated (*A* and *C*) and insulin-treated cells with (*B* and *D*). Experiments were identical to Fig. 4 except POA was added 5 min prior to re-addition of 20 mm Ca^2+^. *E*, mean % relative clearance (± S.E.) of corresponding time-matched control (*white box*), insulin treatment alone (*light gray box*), POA treatment (50 and 100 μm; *black box*), and POA with insulin (*dark gray box*). *, *p* < 0.05; **, *p* < 0.01 compared with corresponding time-matched control as determined by Kurskal Wallis with post hoc Dunns test.

**FIGURE 7.**
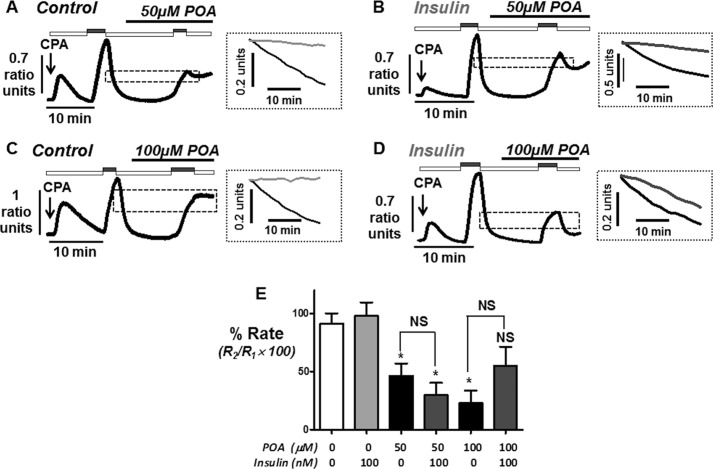
**Insulin protection against palmitoleic-induced inhibition of the PMCA during long (10 min) *in situ* [Ca^2+^]*_i_* clearance assays.** Representative traces showing the effect of POA (*A–D*) on *in situ* [Ca^2+^]_i_ clearance in untreated (*A* and *C*) and insulin-treated cells with (*B* and *D*). Experiments were identical to Fig. 4 except POA was added 10 min prior to re-addition of 20 mm Ca^2+^. *E*, mean % relative clearance (± S.E.) of corresponding time-matched control (*white box*), insulin treatment alone (*light gray box*), POA treatment (50 and 100 μm; *black box*), and POA with insulin (*dark gray box*). *, *p* < 0.05; **, *p* < 0.01 compared with corresponding time-matched control as determined by Kurskal Wallis with post hoc Dunns test.

Because [Ca^2+^]*_i_* clearance was well maintained for up to 10 min between consecutive influx-clearance phases, the effect of POA treatments (50 and 100 μm) over these different time periods (2, 5, and 10 min) was further tested with or without insulin treatment (100 nm for 30 min). As previously mentioned, over 2 min 50 μm POA had no significant effect on [Ca^2+^]*_i_* clearance either without (99 ± 2%, *n* = 5; see Fig. 5, *A* and *E*) or with insulin treatment (101 ± 6%, *n* = 8; see Fig. 5, *B* and *E*). However, 100 μm POA caused a significant inhibition to 59 ± 6% (*n* = 6, *p* < 0.05; Fig. 5, *C* and *E*), which was completely prevented in insulin-treated cells (105 ± 6%, *n* = 6, *p* < 0.05; Fig. 5, *D* and *E*). Over 5 min 50 μm POA now caused a significant inhibition of [Ca^2+^]*_i_* clearance to 64 ± 8% (*n* = 6, *p* < 0.05; Fig. 6, *A* and *E*), which was completely prevented by insulin treatment (124 ± 11%, *n* = 6, *p* < 0.05; Fig. 6, *B* and *E*). Similarly 100 μm POA caused an even greater inhibition of [Ca^2+^]*_i_* clearance over 5 min (28 ± 10%, *n* = 6, *p* < 0.05; Fig. 6, *C* and *E*), which was also completely prevented in insulin-treated cells (114 ± 9% *n* = 6, *p* < 0.05; Fig. 6, *D* and *E*). Finally, over 10 min the inhibitory effects of POA were so severe (50 μm POA, 46 ± 11%, *n* = 6, *p* < 0.05; Fig. 7, *A* and *E*; 100 μm POA, 23 ± 11%, *n* = 6, *p* < 0.05; Fig. 7, *C* and *E*), that insulin failed to exhibit any protection (50 μm POA, 30 ± 11%, *n* = 6, *p* < 0.05; Fig. 7, *B* and *E*; 100 μm POA, 55 ± 6%, *n* = 6, *p* < 0.05; Fig. 7, *D* and *E*). However, these responses were highly variable, partly due to the fact that Ca^2+^ influx was also markedly inhibited, making analysis very difficult. Nevertheless, there was clearly a lack of protection by insulin under these conditions, suggesting that such a dramatic inhibition of Ca^2+^ clearance, regardless of any effect on Ca^2+^ influx, was so severe that insulin was unable to overcome this effect.

**FIGURE 8.**
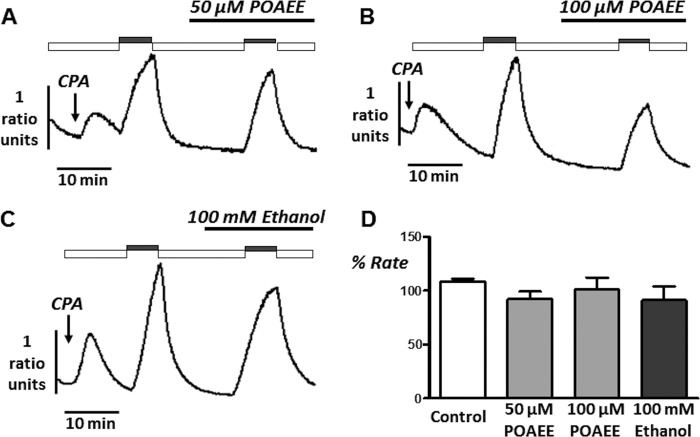
**Ethanol and the non-oxidative fatty acid/ethanol metabolite, POAEE has no effect on PMCA activity during *in situ* [Ca^2+^]*_i_* clearance experiments.** Representative traces showing the effect of POAEE (50 μm, *A* and 100 μm, *B*) and 100 mm ethanol (*C*) on *in situ* [Ca^2+^]*_i_* clearance. Experiments were identical to Fig. 4 except POAEE was added 10 min prior to re-addition of 20 mm Ca^2+^. *D*, mean % relative clearance (± S.E.) of corresponding time-matched control (*white box*), POAEE (*gray box*), and ethanol treatment (*black box*).

Collectively these data provide evidence that insulin protects the PMCA from inhibition by pancreatitis-inducing agents such as POA. This likely explains, at least in part why insulin prevents POA-induced [Ca^2+^]*_i_* overload. It is also worth noting that neither ethanol (as high as 100 mm) nor POAEE (as high as 100 μm), when applied for up to 10 min prior to the second influx-clearance phase had any effect on the Ca^2+^ clearance rate (Fig. 8). This further reinforces the notion that POA is the major cytotoxic pancreatitis-inducing agent.

##### Insulin Attenuates the POA-induced ATP Depletion

**FIGURE 9.**
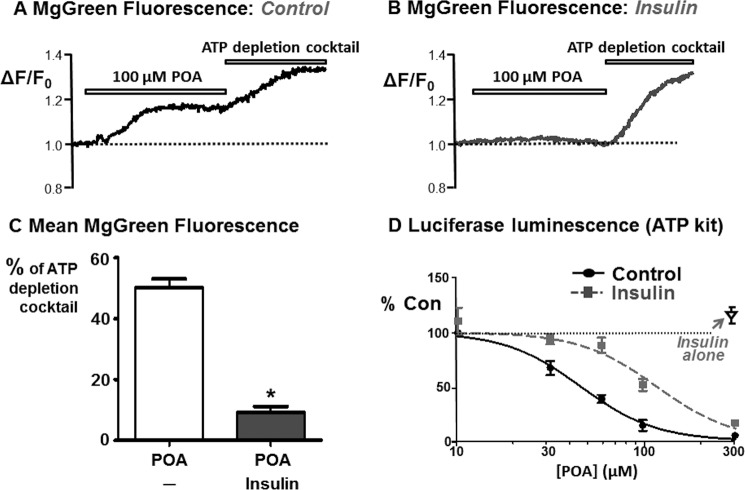
**Insulin attenuates POA-induced ATP depletion.** Cellular ATP was assessed using MgGreen fluorescence (*A--C*) and the *in vitro* ViaLight Plus luciferase-based chemiluminescence ATP monitoring kit (*D*). Representative traces showing the relative MgGreen fluorescence (F/F_0_) in response to 100 μm POA followed by the “ATP depletion” mixture in untreated control cells (*A*) and insulin-treated cells (*B*). *C*, responses to POA were quantified and normalized to the ATP depletion mixture response and compared between untreated control cells and insulin-treated cells (*, *p* < 0.05, as assessed using a Mann Whitney *U* test). *D*, average POA concentration-ATP depletion response curves for untreated control cells (*black circles*) and cells pre-treated with 100 nm insulin (*gray squares*), measured using the ATP kit. Pancreatic acinar cells were treated with or without 100 nm insulin for 20 min, followed by treatment with or without various concentrations of POA (10–300 μm) for a further 20 min. Luminescence was normalized to the corresponding time-matched control (total ATP) and additional control experiments were performed in which cells were treated with 100 nm insulin alone (*open triangle*).

POA has been previously shown to induce mitochondrial depolarization, impair mitochondrial function and thus induce ATP depletion in pancreatic acinar cells (24, 25, 32, 33). Since insulin abolished the POA-induced [Ca^2+^]*_i_* overload and inhibition of the PMCA, we next wanted to investigate whether this was due to preservation of ATP, similar to our previous study with H_2_O_2_ (22). This was investigated using two complementary approaches; MgGreen fluorescence in intact living cells and *in vitro* chemiluminescence assays of fire-fly luciferase (22, 30). MgGreen was used to assess ATP depletion indirectly by measuring free [Mg^2+^], as in our previous studies (30). Using the MgGreen technique, 100 μm POA caused 50 ± 3% ATP depletion (Fig. 9, *A* and *C*, *n* = 4). However, following treatment with 100 nm insulin, the response to 100 μm POA was markedly reduced to 9 ± 2% ATP depletion (Fig. 9, *B* and *C*, *n* = 4).

For experiments using the *in vitro* fire-fly luciferase chemiluminescence ATP assays, the experimental design was essentially the same as our previous study with H_2_O_2_ (30). These experiments revealed that POA caused a steep concentration-dependent ATP depletion between 10 μm, at which there was no significant ATP depletion, and 300 μm, which reached close to maximum ATP depletion (see Fig. 9*D*). The data were fitted to log transformed sigmoidal concentration-response curves which generated an average IC_50_ of 46 ± 1.1 μm and Hill slope of 2.1 ± 0.2 (*n* = 5 assays, 3 rats; Fig. 9*D*, *black circles*). Pre-treatment of cells with insulin (100 nm) caused a rightward shift in the concentration-response curve and significantly increased the average IC_50_ to 119 ± 1.1 μm (*p* < 0.05; Hill slope of 2.1 ± 0.5; *n* = 5 assays, 3 rats; Fig. 9*D*, *gray squares*). Surprisingly, insulin treatment alone (without POA) for the entire experimental period (3 × 20 min) significantly increased ATP levels compared with time-matched controls (115 ± 5%; *n* = 5 assays, 3 rats; Fig. 9*D*, *open triangle*; *p* < 0.001 as assessed by a one sample *t* test). Using both MgGreen and *in vitro* luciferase assays, these data suggest that insulin protects acinar cells from substantial ATP depletion induced by POA over the relatively short time period of 15–20 min.

##### Insulin Attenuates POA-induced Necrosis

**FIGURE 10.**
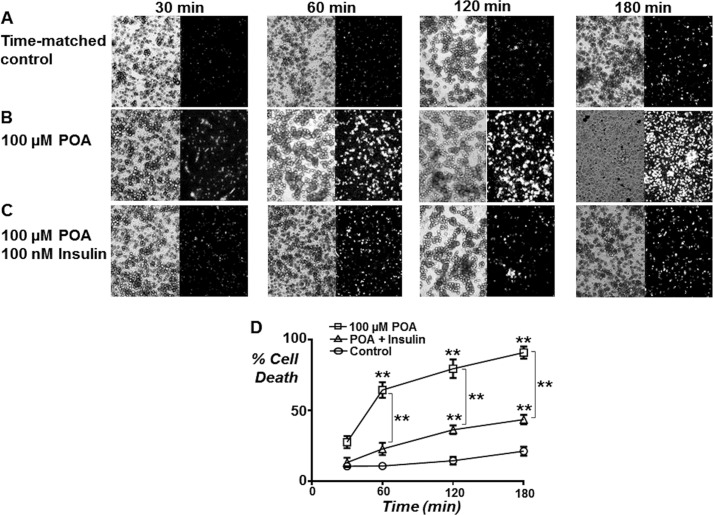
**Insulin attenuates the POA-induced necrosis.** Necrotic cell death was assessed using the cell impermeant fluorescent nuclear-staining dye, PI. Cells were stained with 1.5 μm PI for 30 min prior to the end of each treatment period. Representative images of brightfield (*left*) and corresponding PI fluorescence for time-matched control (*A*) or following treatment with 100 μm POA (*B*) or a combination of 100 μm POA and 100 nm insulin (*C*) for 30, 60, 120, and 180 min. PI-positive cell count was normalized to total cell count from brightfield images and mean % cell death (± S.E.) plotted over treatment period (*D*). *, *p* < 0.05; **, *p* < 0.01 compared with corresponding time-matched control as determined by two-way ANOVA with post hoc Bonferroni correction.

Finally, we wanted to test the pathophysiological implication of insulin's protection on POA-induced necrotic cell death using propidium iodide (PI). PI is a cell impermeable nuclear-staining fluorescent dye that will only stain cell nuclei that have undergone cell lysis as a consequence of necrosis. Cell death was quantified by normalizing the PI-positive cell count to the total number of cells in the corresponding brightfield image (% cell death). In untreated time-matched control cells % necrotic cell death increased slightly from 10.4 ± 2.4% at 30 min to 21.1 ± 3.2% at 180 min (Fig. 10). This relatively high basal cell death even at 30 min was likely due to the inherent nature of the cell isolation procedure producing a high proportion of cellular injury. Treatment with 100 μm POA significantly increased cell death to 64.4 ± 5.6% after only 60 min (*p* < 0.01), which continued to increase further at 120 min (79.6 ± 6.6%) and 180 min (91.1 ± 4.3%, *n* = 5, *p* < 0.01; Fig. 10). It was also clear that the cells had undergone cell lysis from the corresponding brightfield images especially at 180 min (Fig. 10). However, pre-treatment with insulin (100 nm) dramatically and significantly attenuated the POA-induced cell death at 60 (22.7 ± 4.4%), 120 (36.3 ± 3.1%), and 180 min (43.5 ± 3.2%, *n* = 5, *p* < 0.01; Fig. 10). These data provide strong evidence that insulin prevents necrosis induced by *bona fide* pancreatitis-inducing agents, such as POA.

## DISCUSSION

Excessive alcohol consumption and specifically fatty acid/ethanol metabolites is a major cause of pancreatitis (1–3). Ethanol is thought to combine with saturated fatty acids, such as palmitic acid (PA), within the pancreas and is converted to POAEE, by POAEE synthase and acyl-coenzyme A: ethanol *O*-transferase (26). For many years POAEE was thought to be the major cytotoxic biproduct of fatty acid/ethanol metabolism during alcohol-induced pancreatitis. This is because POAEE accumulates in the pancreas of alcoholic pancreatitics (27). Moreover, POAEE has been reported to induce [Ca^2+^]*_i_* oscillations but the more cytotoxic effects are thought to be mediated by the metabolism of POAEE to POA by carboxylesterase (26). This is because inhibition of the carboxylesterase with bis-(4-nitrophenyl)phosphate (BNPP) abolished the POAEE-induced necrosis but not the POAEE-induced [Ca^2+^]*_i_* oscillations (24). Therefore, POA is likely responsible for pancreatitis induced by excessive alcohol consumption. Indeed results of the present study show that POA was the most cytotoxic of these pancreatitis-inducing agents. Specifically, POA induced ATP depletion, inhibition of the PMCA, irreversible Ca^2+^ overload and the consequent necrotic cell death, consistent with previous studies (24, 25). POAEE and ethanol on the other hand caused only minor changes in Ca^2+^ and had no effect on PMCA activity, even over prolonged periods. This therefore reinforces the notion that POA is the major cytotoxic by-product of fatty acid/ethanol metabolism during alcohol-induced pancreatitis (24–29).

The nature of the POA-induced increase in [Ca^2+^]*_i_* was slow, sluggish, and reminiscent of a thapsigargin or CPA response and thus inhibition of SERCA. POA is reported to induce mitochondrial depolarization and ATP depletion in pancreatic acinar cells (24, 25). Therefore, the most likely mechanism for the POA-induced [Ca^2+^]*_i_* overload is inhibition of SERCA (and PMCA), store depletion and the activation of store-operated Ca^2+^ entry. However, it is important to note that these POA-induced [Ca^2+^]*_i_* responses were nearly always irreversible and subsequent stimulation with CCK had no effect. Although we cannot rule out an effect on SERCA and SOCE as a source of the increase in [Ca^2+^]*_i_*, the irreversible nature of these responses suggests that PMCA activity is also most likely severely inhibited. In the context of pancreatic acinar cell injury inhibition of the PMCA is likely more important than inhibition of SERCA. This is because inhibition of the PMCA, regardless of whether the SERCA is inhibited, will always induce an irreversible Ca^2+^ overload in pancreatic acinar cells. This is because the ER has a finite capacity to store Ca^2+^ whereas the PMCA pumps Ca^2+^ into an effectively infinite extracellular compartment. Moreover, the PMCA is the only Ca^2+^ efflux pathway, due to the lack of NCX expression (34). On the other hand, if the PMCA remains functional even in the face of impaired SERCA activity, [Ca^2+^]*_i_* will nearly always recover to baseline; for example, as seen with the SERCA inhibitors, thapsigargin or CPA. Moreover, the PMCA is regarded as critical in maintaining low resting [Ca^2+^]*_i_* below 300 nm, due to its high affinity for Ca^2+^ (35). For this reason we decided to focus our investigation on PMCA activity, rather than SERCA, using our *in situ* [Ca^2+^]*_i_* clearance assay. These experiments revealed that POA induced both a concentration and time-dependent inhibition of PMCA activity, whereas POAEE and ethanol had no effect even when applied over prolonged periods (up to 10 min). Therefore, protection of the PMCA might be an important therapeutic strategy for preventing Ca^2+^ overload and the consequent acinar cell necrosis.

The most significant finding from the current study is that insulin treatment effectively abolished the POA-induced ATP depletion, inhibition of the PMCA, Ca^2+^ overload, and necrosis. The latter has major pathophysiological implications because pancreatic necrosis leads to a spiral of self-perpetuating tissue injury and systemic inflammation culminating in multiple organ failure (1–5). Although these findings are novel, several lines of evidence from animal studies and human clinical studies suggest that insulin might have a protective role during pancreatitis (9, 12, 13, 16, 18–21, 36–43).

In streptozotocin (STZ)-induced diabetic mice, in which insulin secretion is impaired, caerulein-induced acute pancreatitis is much worse than in control mice (16). However, this was prevented by exogenously applied insulin (16). In l-arginine-induced pancreatitis, acinar cells surrounding the islets of Langerhans remain relatively intact compared with distal injured acinar cells suggesting a paracrine protective role for insulin (12, 13).

In human pancreatitis, treatment is largely restricted to nutritional support, often accompanied by insulin therapy to reduce hyperglycemia-associated sepsis (21) and systemic inflammation (19, 20). Insulin is also used clinically to treat hypertriglyceridemia-induced pancreatitis (43). Moreover, there are strong links between diabetes/hyperglycemia and the severity of pancreatitis. Diabetes increases the mortality in patients with chronic pancreatitis (37, 38) and exacerbates exocrine pancreatic injury (40). Patients with diabetes are at higher risk of developing pancreatitis (18, 36, 41, 42) and pancreatitis patients with hyperglycemia are at higher risk of multiple organ failure (39). Furthermore, in a large scale population-based study the incidence of acute pancreatitis was markedly reduced among insulin-treated diabetic patients (9). However, in all these clinical studies it is very difficult to separate the systemic effects of inflammation or hyperglycemia-associated sepsis, which may occur during diabetes, from the loss of any direct protective effects of insulin within the pancreas. Likewise, it is unclear whether exogenous insulin therapy prevents these systemic effects or directly protects acinar cells from cellular injury. However, the current study provides the first evidence that insulin directly protects acinar cells againist *bona fide* pancreatitis-inducing agents in cellular models of the disease.

In our previous study the protective effects of insulin were due to activation of PI3K/Akt and a switch from mitochondrial metabolism toward glycolysis as the major source of ATP to fuel the PMCA (22). However, the current study shows that the protective effects of insulin are only partly due to activation of PI3K/Akt and that a PI3K-independent effect may also be partly responsible. The nature of the PI3K-independent effects of insulin are much less well understood even in classically insulin-sensitive tissues, such as muscle, fat, and liver cells, and almost completely unknown in tissues such as pancreatic acinar cells. Candidates include MAPK and tyrosine kinase pathways. Although MAPK has many downstream targets, most are involved in transcriptional control and thus unlikely to be responsible for acute regulation of metabolism and specifically glycolytic flux (44). The insulin receptor has intrinsic tyrosine kinase activity and therefore tyrosine phosphorylation of key glycolytic enzymes may be a potential mechanism responsible for the protective effects of insulin. However, dissecting this putative mechanism pharmacologically is not trivial. This is because tyrosine kinase-mediated phosphorylation of the PMCA is reported to inhibit PMCA activity (45). Therefore, tyrosine kinase inhibitors might be expected to increase PMCA activity independently of any effect on metabolism.

Another possible explanation for the protective effects of insulin is that insulin activates insulin-like growth factor receptors (IGF-1Rs), especially at 100 nm concentration. Indeed IGF-1Rs are expressed in pancreatic acinar cells and activate similar downstream signaling pathways (46). This concentration of insulin (100 nm), which is much higher than circulating concentrations (50–350 pm) (47), was chosen as it evoked the best cellular protection in all responses tested. In pancreatic acinar cells studies show that insulin binds to the insulin receptor (IR) with a *K_d_* of 2 nm (high affinity site) and 88 nm (low affinity site) (48). This suggests that 100 nm insulin is within the physiological range for maximum activation of IRs in acinar cells. Crucially, insulin is reported to bind to IGF-1Rs on pancreatic acini with ∼1000 fold lower affinity (IC_50_ for IGF-1 ∼0.5 nm, IC_50_ for insulin ∼0.5 μm) (49). This suggests that, even at 100 nm, the protective effects of insulin are most likely mediated through IRs and not IGF-1Rs. It is also worth noting that the local concentration of insulin at the site of release from β-cells within the pancreas, and thus close to acinar cells, may be much higher than circulating insulin (∼10 nm) (50).

As alluded to above, ATP depletion is likely to affect both SERCA and PMCA; however, the irreversible nature of the POA-induced Ca^2+^ overload response suggests that PMCA may be more important. We therefore focused our investigation of the protective effects of insulin on PMCA activity. As expected, and consistent with the resting [Ca^2+^]*_i_* data, POA-induced inhibition of the PMCA was almost entirely abolished by insulin pre-treatment. This was certainly true when POA was applied for up to 5 min prior to the second influx-clearance phase. However, over longer periods of POA treatment inhibition of the PMCA appeared to be so “severe” that pre-treatment with insulin failed to overcome this inibition. This temporal effect of both POA-induced inhibition of the PMCA and protection by insulin could be explained by the rate and extent of ATP depletion induced by POA that is sufficient to inhibit the PMCA. Using MgGreen to dynamically assess ATP depletion, it took up to 15 min for POA to reach the maximum MgGreen response, which was never as much as the ATP depletion induced by the mixture. In addition, using the luciferase assay to assess ATP depletion, although insulin shifted the concentration-ATP depletion response curve to the right, there were clearly higher concentrations of POA where insulin failed to have any protective effect. These data therefore suggest that insulin pre-treatment preserves cellular ATP to some extent during POA-induced cellular stress. Regardless of the absolute ATP concentration, this seems to be sufficient to maintain PMCA function and prevent the deleterious effects of cytotoxic Ca^2+^ overload.

Interestingly, insulin prevented the apparent “run-down” of PMCA activity during the long (20 min) time-matched control *in situ* [Ca^2+^]*_i_* clearance assays. This is not surprising as the cells are likely to be under considerable stress under the conditions of these experiments. Moreover, the PMCA is a major consumer of cellular ATP (51, 52), therefore, over prolonged periods ATP may well start to fall resulting in “run-down” of the PMCA. However, this appears to be circumvented by insulin treatment, which can be assumed to be due to preservation of ATP.

Our previous study showed that insulin switches metabolism from mitochondrial metabolism toward glycolysis (22). This appears to maintain ATP sufficiently to fuel PMCA even in the face of impaired mitochondrial function (22). The specific mechanism for this insulin-mediated increased glycolytic ATP remains unclear, but likely involves either Akt or tyrosine kinase-mediated phosphorylation of key glycolytic enzymes or regulatory proteins. However, when one considers the numerous isoforms and splice variants of each enzyme in the glycolytic pathway, not to mention additional regulatory proteins, the number of potential candidates becomes vast. Therefore, identification of the specific molecular mechanism for the effects of insulin is beyond the scope of this study and will be the focus of future investigations.

Nevertheless, there are a few primary candidates. Phosphofructokinase-1 (PFK1) converts fructose-6-phosphate to fructose-1,6-bisphosphate and represents the major rate-limiting step in glycolysis. PFK-1 can be activated by insulin via tyrosine kinase-mediated phosphorylation (53). Phosphofructokinase-2 (PFK2) converts fructose-6-phosphate to fructose-2,6-bisphosphate, a potent allosteric activator of PFK1. Insulin can also activate PFK2, via Akt-mediated phosphorylation, which in turn activates PFK1 and thus total glycolytic flux (54). Furthermore, in red blood cells glycolytic enzymes associate with the plasma membrane, which if extrapolated to pancreatic acinar cells, suggests that increased glycolytic flux by insulin may provide a privileged ATP supply to the PMCA (55–59). Indeed functional studies of ^45^Ca^2+^ flux (and thus PMCA activity) into isolated inside-out plasma membrane vesicles from pig stomach smooth muscle cells showed that the PMCA has its own glycolytic ATP supply (60, 61). This is unlikely to be a limiting factor when global cellular ATP is high and thus well above the saturating concentration for the PMCA (*i.e.* >1 mm). However, in the face of impaired mitochondrial metabolism a “privileged” glycolytic ATP supply to the PMCA may become critical for maintaining low resting [Ca^2+^]*_i_* and thus avoiding stress-induced necrosis.

In summary, the current study combined with our previous study provide compelling evidence that insulin has a direct protective effect on pancreatic acinar cells. This therefore suggests that insulin therapy with tight glycemic control, for example using the hyperinsulinaemic-euglycemic clamp, might be an effective treatment for severe acute pancreatitis. Moreover, the current study provides a strong rationale for further studies dissecting the molecular mechanism for the insulin-mediated protection of the PMCA in pancreatic acinar cells so that more specific drugs can be designed to treat pancreatitis without the deleterious effects of inadvertent hypoglycemia from insulin therapy.

## Footnotes

4 The abbreviations used are:

AP: acute pancreatitis
[Ca^2+^]*_i_*: intracellular Ca^2+^ concentration
PMCA: plasma membrane Ca^2+^-ATPase
H_2_O_2_: hydrogen peroxide
NCX: Na^+^-Ca^2+^ exchange
POA: palmitoleic acid
POAEE: palmitoleic acid ethylester
TMC: time-matched control
AUC: area under the curve
CPA: cyclopiazonic acid
SERCA: sarco/endoplasmic reticulum Ca^2+^-ATPase
SOCE: store-operated Ca^2+^ entry
CCD: charged-coupled device
BNPP: bis-(4-nitrophenyl)phosphate.
